# Effect of Kangshuanyihao Formula on the Inflammatory Reaction and SIRT1/TLR4/NF-*κ*B Signaling Pathway in Endothelial Injury

**DOI:** 10.1155/2017/9019765

**Published:** 2017-04-30

**Authors:** Jie Han, Hua-Qin Tong, Song-Yi Cheng, Li Yang, Han-Yu Chen, Jian-Dong Chen, Xiao-Hu Chen

**Affiliations:** ^1^First Clinical Medical College, Nanjing University of Chinese Medicine, Nanjing 210023, China; ^2^Department of Cardiology, Jiangsu Province Hospital of Traditional Chinese Medicine, The Affiliated Hospital of Nanjing University of Chinese Medicine, Nanjing 210029, China

## Abstract

Endothelial injury plays an important role in atherosclerosis (AS). Kangshuanyihao formula uses therapeutic principles from Chinese medicine to supplement Qi, thereby promoting blood circulation, and remove blood stasis. The mechanism by which the formula inhibits endothelial injury was examined in a rat model of 1,25-dihydroxyvitamin D_3_ (VD_3_) intraperitoneal injection and high-fat-induced endothelial injury. Rats were randomly divided into the model, high-dose, middle-dose, low-dose, positive drug (rosuvastatin), and combination (positive drug + middle-dose) groups; 10 Sprague-Dawley rats served as the blank group. The aortic endothelium was stained with hematoxylin and eosin and the levels of blood lipids and inflammation markers (mRNA and protein) were measured. Endothelial injury, lipid levels, and inflammation were increased in the model. Kangshuanyihao formula reduced endothelial injury, improved lipid levels, and downregulated inflammation, as shown by significant reduction of the protein levels of SIRT1, TLR4, and NF-*κ*B and mRNA levels of SIRT1, TLR4, NF-*κ*B, IL-1*β*, IL-6, and IL-12. Thus, we conclude that Kangshuanyihao formula can inhibit the inflammatory reaction in the rat model of high-fat-induced endothelial injury after intraperitoneal injection of VD_3_. This mechanism may be attributed to regulating the SIRT1/TLR4/NF-*κ*B signaling pathway.

## 1. Introduction

Cardiovascular diseases, with high morbidity and mortality [1], have become the most chronic cause of death worldwide [2]. Nowadays, we have considered that atherosclerosis (AS) is the basis of cardiovascular diseases; therefore, it is essential to prevent and treat AS for the better prognosis of patients with cardiovascular diseases.

AS, which plays an important role in cardiovascular diseases, is usually caused by vascular endothelial injury [3], which affects the development of this chronic inflammatory disease. After many years of research, the pathogenesis of AS is still not entirely clear, and various factors are involved in the occurrence of AS, including lipid levels, inflammation, platelet involvement, and endothelial cell injury. Research has shown that the progression of AS starts with vascular endothelial injury and lipid infiltration, which are influenced by the inflammatory response [4, 5]. In the inflammatory mechanism, it was found that nuclear factor-*κ*B (NF-*κ*B) is an important transcription factor that initiates the expression of proinflammatory cytokines in the inflammatory response. NF-*κ*B is a cell transcription factor that plays an important role in the regulation of many immune and inflammatory responses [6]. NF-*κ*B activation, by promoting the expression of inflammatory factors such as a series of chronic inflammatory processes, results in increased endothelial injury and lipid deposition, thereby promoting the formation and progression of AS [7, 8].

Kangshuanyihao formula, which comprises Sumu* (Caesalpinia)*, Honghua (safflower), and Shuizhi (leeches) and promotes Qi by activating blood as a basic treatment, is summed up in clinical experience. Modern research has shown that the ethyl acetate extract of* Caesalpinia sappan* can promote blood circulation to remove blood stasis and can restrain inflammation [9]. Several studies have also shown that safflower's main components have the effect of lowering blood lipid levels and inhibiting the progression of AS [10–12]. Leeches can also lower blood cholesterol levels and plaque lipid levels and can effectively reduce the levels of the inflammatory factor tumor necrosis factor-*α* (TNF-*α*) [13–15].

In this study, we used this formula to observe whether it can play a role in treating vascular endothelial injury. This study was designed to investigate this formula's function to lower the lipid levels of rats by detecting the levels of total cholesterol (TC), triglyceride (TG), low-density lipoprotein cholesterol (LDL-C), and high-density lipoprotein cholesterol (HDL-C) and control the development of inflammation by detecting the expression levels of TNF-*α*, interleukin- (IL-) 1*β*, IL-6, and IL-12. We also investigated the inflammatory mechanism to verify whether the formula can inhibit the formation of AS to identify effective drugs that individually control AS or that can be used to control AS combined with other drugs.

## 2. Materials and Methods

### 2.1. Animals

Normal male Sprague-Dawley (SD) rats were purchased from Beijing Vital River Laboratory Animal Technology Co., Ltd. SD rats were raised in a specific pathogen-free environment at a room temperature of 22°C to 24°C, 40%–50% relative humidity, and a 12-hour light/dark cycle. The procedures were performed in accordance with the National Institutes of Health's Guide for the Use and Care of Laboratory Animals and were approved by the Committee on Animal Care. SD rats weighing 185–225 g (*n* = 60) were chosen for the endothelial injury model established by intraperitoneal injection of 1,25-dihydroxyvitamin D_3_ (VD_3_) with a high-fat diet; also there are 10 rats fed with normal diet.

### 2.2. Animal Model of Endothelial Injury

Rats were fed for one week to adapt to the environment and were modeled by the intraperitoneal injection of VD_3_ and a high-fat diet. After one week of normal feeding, the animals treated with 600,000 U/kg of VD_3_, and 100,000 U/kg of VD_3_ in the 3rd, 6th, and 9th weeks were fed for 9 weeks with a high-fat diet.

### 2.3. Medications and Grouping

Kangshuanyihao formula was composed of ingredients such as* Caesalpinia sappan* Linn.,* Carthamus tinctorius* L., and* Whitmania pigra* Whitman. The formula was produced by Jiangyin Pharmaceutical Factory, and the final concentration was 0.5 g of crude drug/mL. Lipid kits were purchased from Nanjing Jiancheng Bioengineering Institute (Art. numbers A113-1, A112-1, A111-1, and A110-1). Enzyme-linked immunosorbent assay (ELISA) kits for the detection of IL-1*β*, IL-2, IL-6, and TNF-*α* were purchased from Nanjing Jiancheng Bioengineering Institute (Art. numbers H002, H007, H052, and H010). Sirtuin 1 (SIRT1) (ab110304), Toll-like receptor 4 (TLR4) (ab8376), and NF-*κ*B (ab16502) antibodies, as well as the secondary antibodies, were purchased from Abcam.

After the intraperitoneal injection of VD_3_ and feeding with a high-fat diet, the rats were randomly divided into 6 groups of 10 rats each: the model, high-dose, middle-dose, low-dose, positive drug (i.e., rosuvastatin), and combination (positive drug + middle-dose) groups. The other 10 SD rats were used as the blank group (B). The dosages of rosuvastatin and Kangshuanyihao formula, which were based on the clinical daily dosages for adult humans with a dose conversion coefficient, were as follows: Kangshuanyihao formula groups: 1 g/kg/d (corresponding to 12 times the clinical dosages), 2 g/kg/d, and 4 g/kg/d; rosuvastatin group: 2 mg/kg/d (12 times the clinical dosages). The blank group received distilled water at 5 mL/kg/d. The medicine was dissolved in distilled water, and the medicine was administered to the rats using gastric lavage once daily for 28 days.

### 2.4. HPLC

*Preparation of Standard Solutions*. A mixed standard stock solution containing uridine, inosine, guanosine, safflomin A, and brazilin was prepared in MeOH/H_2_O (50 : 50, v/v). The standard solutions were filtered through a 0.22 *μ*m membrane prior to injection. All solutions were stored in a refrigerator at 4°C before analysis.

*HPLC Analysis of Sample*. Analysis was performed on Waters 2695 Alliance HPLC system (Waters Corp., Milford, MA), consisting of a quaternary pump solvent management system, an online degasser, and an autosampler. The raw data were detected by Waters 2998 PDA, acquired, and processed with Empower Software. A Waters X-select C18 column (5 *μ*m, 4.6 mm × 250 mm) preceded by a RP C_18_ guard column (5 *μ*m, 3.9 mm × 20 mm) was applied for all chromatographic separation. Detection wavelength was set at 260 nm for all compounds. The mobile phase was composed of A (methanol) and B (0.1% aqueous ethylic acid) with a linear gradient elution: 0–20 min, 5% A; 20–75 min, 5–50% A, then keeping 50% A for 10 min to clean the column. MeOH/H_2_O (10 : 90, v/v) were used as solutions for cleaning the injection needle. The flow rate was set at 0.80 mL/min and the injection volume was 10 *μ*L. The column temperature was maintained at 30°C.

### 2.5. Histology

All of the rats in the seven groups were euthanized with 0.1% pentobarbital sodium. When the blood was collected from the abdominal aorta, the later was quickly removed, fixed in 10% formaldehyde, embedded, and sectioned to determine the morphology of any atherosclerotic plaque by hematoxylin and eosin (HE) staining.

### 2.6. Immunohistochemistry

The chips were first baked in a 60°C incubator for 60 minutes before dewaxing and then were dewaxed by soaking in xylene for 10 minutes twice. Thereafter, the sections were soaked in absolute ethanol, 95% ethanol, 85% ethanol, and 70% ethanol for 5 minutes each before phosphate-buffered saline (PBS) washing. Next, the antigen was reconstituted with antigen-repair solution and was washed three times with PBS. The sections were then treated with 3% H_2_O_2_-methanol solution for 15 minutes to inactivate the enzyme followed by washing with PBS. 100 mL of primary antibody (1 : 100) was added and incubated in a humidified chamber at 37°C for 2 hours. Next, 50 *μ*L of the potentiator was added and incubated in a humidified chamber at room temperature for 20 minutes. Fifty microliters of HRP-labeled secondary antibody was then added at room temperature, followed by incubation for 30 min at 37°C. The sections were washed with PBS between each step, and the color was stained with two drops of freshly prepared 3,3′-diaminobenzidine solution. The staining depth was observed by microscopy. The staining was stopped immediately, and the color reaction was terminated with distilled water for 15 minutes. The sections were stained with hematoxylin for 30 minutes and were rinsed with distilled water. The sections were then placed into the hydrochloric acid-methanol solution and were immediately rinsed with distilled water. Thereafter, they were immersed in 70%, 85%, 95%, and absolute ethanol for 5 minutes each. They were then soaked for 10 minutes in xylene, followed by replacement of the xylene and soaking for another 10 minutes. After drying, neutral gum was added to the sections, followed by the addition of coverslips. Finally, the sections were observed using an optical microscope.

### 2.7. Lipid Level Determination

The animals were killed, and blood samples were collected from the aorta. The serum of the animals was separated. Next, the TC, TG, LDL-C, and HDL-C levels were determined by radioimmunoassay according to the manufacturer's instructions: 2.5 *μ*L of distilled water was added to blank wells, 2.5 *μ*L of calibrator (5.17 mmol/L) was added to the standard wells, and 2.5 *μ*L of serum samples was added to each detected hole. Next, 250 *μ*L of working fluid was added to all wells. After mixing for 10 minutes at 37°C, the optical density (OD) values were measured at a wavelength of 510 nm in 10 minutes. The levels were calculated as follows: cholesterol (mmol/L) = [(sample OD − blank OD)/(calibrated OD − blank OD)] × product concentration calibration (5.17 mmol/L).

### 2.8. ELISA Detection

After separation of the serum, we performed ELISA according to the manufacturer's protocol. First, we prepared the reagents, samples, and standards, and then we added the prepared samples and standards, as well as antibodies labeled with enzyme. After allowing the plates to react for 60 minutes at 37°C, they were washed five times, and chromogen solution A and chromogen solution B were added, followed by incubation for 10 minutes at 37°C. In the final step, we added stop solution and measured the OD values, a wavelength of 450 nm in 10 minutes. Next, we used the standard curve prepared with standard concentrations to calculate the concentration of each sample.

### 2.9. Reverse Transcription-Polymerase Chain Reaction (RT-PCR)

The tissue samples from each rat from all the groups were removed and stored at −80°C to examine the mRNA expression. The total RNA from tissue was extracted using the TRIzol kit according to the manufacturer's instructions. cDNA was generated from 500 ng of total RNA using SuperScript II Reverse Transcriptase (Invitrogen). The primers of SIRT1, TLR4, and NF-*κ*B are shown in Table 1. Quantitative reverse transcription-PCR analysis was carried out using SYBR green PCR master mix and was analyzed on a MyIQ real-time PCR cycler (BioRad, Veenendaal, Netherlands). The data were analyzed by the 2^−ΔΔCt^ method. The mRNA level of each sample was normalized to the Ct values of glyceraldehyde 3-phosphate dehydrogenase (GAPDH) amplified from the same sample, ΔCt = Ct_sample_ − Ct_GAPDH_, and the 2^−ΔΔCt^ method was used to the calculate gene expression change.

### 2.10. Western Blotting

Rat tissue proteins were extracted using radio immunoprecipitation assay buffer (25 mM tris-HCl at pH 7.6, 150 mM NaCl, 1% NP-40, 1% sodium deoxycholate, and 0.1% SDS). After complete homogenization on ice rotator, the samples were centrifuged at 12,000*g* for 30 minutes at 4°C to precipitate cell debris. The supernatants were transferred into fresh tubes, and the protein concentrations were determined by the bicinchoninic acid method. The proteins were fractionated by 10% sodium dodecyl sulfate polyacrylamide gel electrophoresis and were electrotransferred onto polyvinylidene fluoride membranes. After blocking with tris-buffered saline containing 5% nonfat milk, the membranes were probed with primary antibody (diluted in accordance with the instructions) at 4°C overnight. Horseradish peroxidase-conjugated secondary antibody was used for luminochemical detection. The band intensities were quantified by densitometry. The results were normalized to that of *β*-actin (1 : 2000 dilution, Abcam).

### 2.11. Statistical Analysis

All of the data were analyzed and expressed using the mean ± standard deviation (*x* ± *s*). Normally distributed data were analyzed using one-way analysis of variance, and the least significant difference was used for comparison between groups. A *P* value < 0.05 was considered to be statistically significant. The SPSS 12.0 software package was used for statistical analysis. In all cases, *P* < 0.05 was deemed to be statistically significant.

## 3. Results

### 3.1. High-Performance Liquid Chromatography Analysis

**Figure 1:**
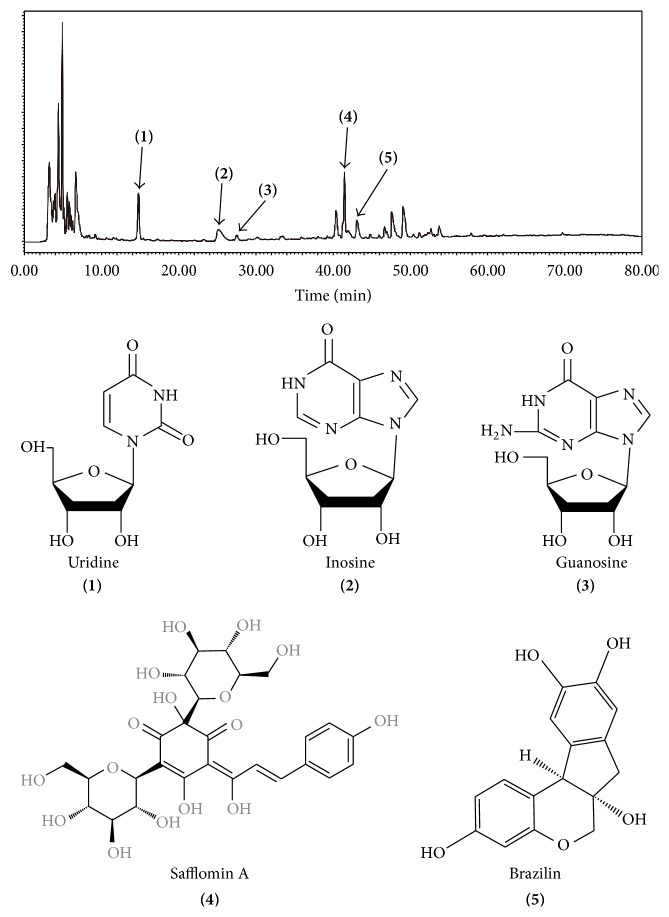
According to the difference in time points, uridine, inosine, guanosine, safflomin A, and brazilin were obtained.

We performed high-performance liquid phase analysis of safflower, hematoxylin, and leeches, and the results are shown in Figure 1. According to the difference in the time points, uridine, inosine, guanosine, safflomin A, and brazilin were obtained. Because the composition of the prescription was relatively simple, it was helpful for further study.

### 3.2. Pathological Staining of the Aorta

**Figure 2:**
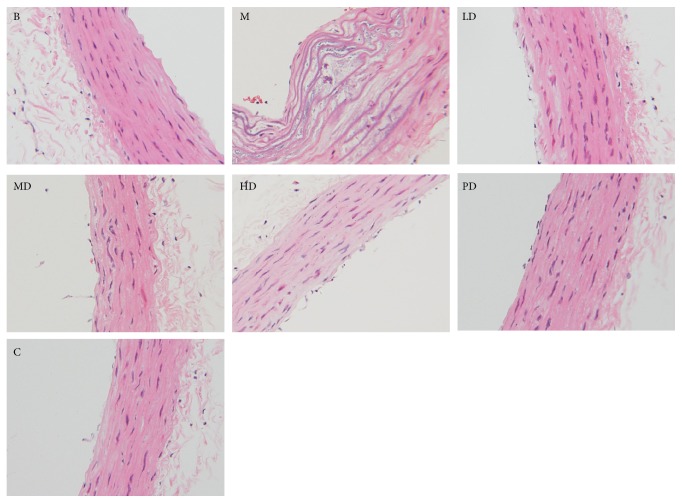
Effects on the rat abdominal aorta (hematoxylin and eosin staining, ×400). (B) Blank group; (M) model group; (HD) high-dose group; (MD) middle-dose group; (LD) low-dose group; (PD) positive drug group; (C) combined group.

After modeling and drug intervention, all of the rats were sacrificed, and then HE staining was carried out on the abdominal aorta of rats. HE staining outcomes showed that, in the model group, the aorta was found to be rough and irregular, endothelial cell arrangement became disordered, and continuity was poor. Additionally, there were a large accumulation of foam cells and proliferation of vascular smooth muscle cells. After drug intervention, the traditional Chinese medicine dose groups and positive drug group demonstrated endometrial arrangement that was more regular and smooth, and only small amount of foam cell accumulation and smooth muscle hyperplasia was evident. The specific results are shown in Figure 2 (400x).

### 3.3. Serum Lipid Levels

**Figure 3:**
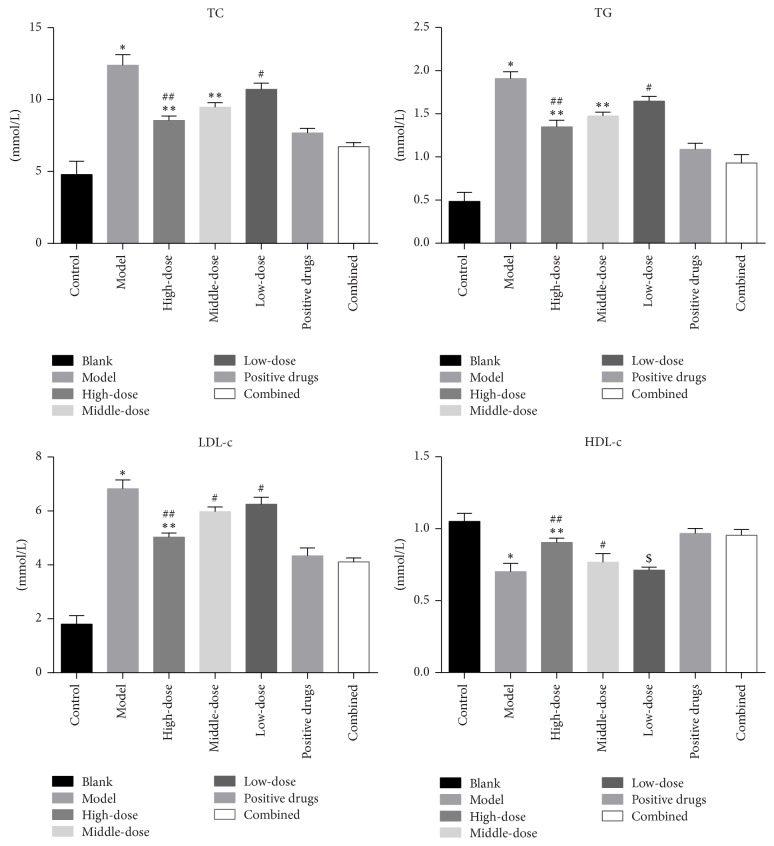
Lipid levels after 4 weeks of intervention in the rats (^*∗*^*P* < 0.01 versus the blank group; ^*∗∗*^*P* < 0.01 versus the model group; ^#^*P* < 0.05 versus the model group; ^##^*P* > 0.05 versus the positive drug group; ^$^*P* > 0.05 versus model group).

After the rats were sacrificed, the serum samples of each group were separated, and the blood lipid levels were measured by radioimmunoassay. Compared with the blank group, the lipid levels were significantly increased in the model groups (*P* < 0.01). Compared with the model group, the levels of TC, TG, and LDL-C in each treatment group were improved (*P* < 0.01 or *P* < 0.05). There was no significant difference between the high-dose and positive drug groups (*P* > 0.05), and the lowering degree was related to the drug doses, as shown in Figure 3.

### 3.4. Inflammatory Factor Levels

**Figure 4:**
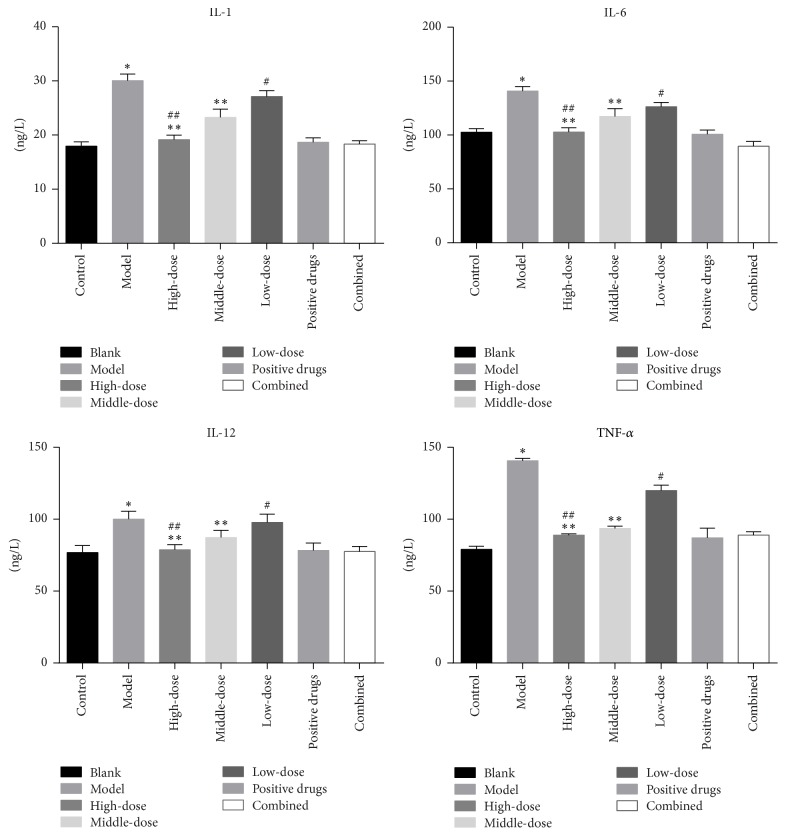
Levels of the inflammation factors after 4 weeks of intervention in the rats (^*∗*^*P* < 0.01 versus the blank group; ^*∗∗*^*P* < 0.01 versus the model group; ^#^*P* < 0.05 versus the model group; ^##^*P* > 0.05 versus the positive drug group).

**Figure 5:**
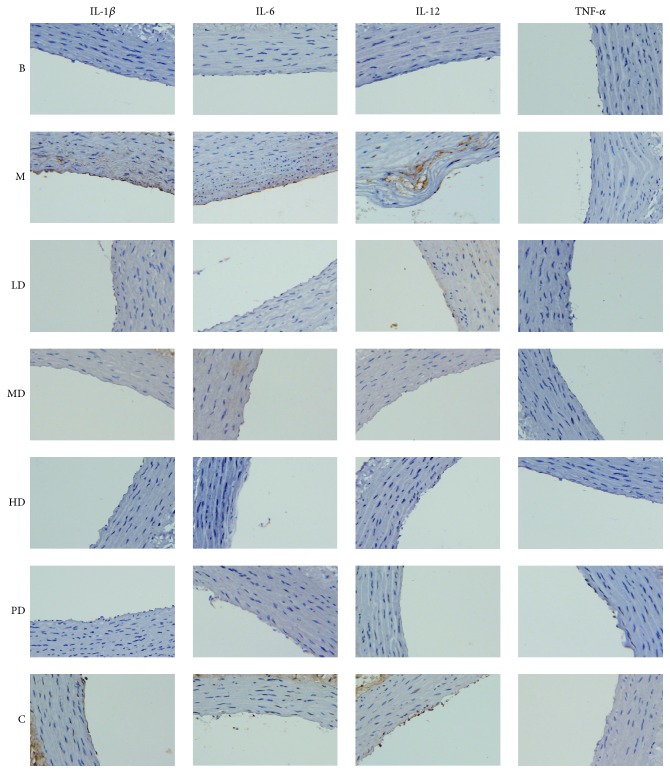
Effects of the rat abdominal aorta (immunohistochemistry, ×400). (B) Blank group; (M) model group; (HD) high-dose group; (MD) middle-dose group; (LD) low-dose group; (PD) positive drug group; (C) combined group.

We used ELISA and immunohistochemistry to carry out the corresponding tests to detect inflammatory factors, and the isolated animal serum samples were analyzed according to the ELISA instructions. The results showed that, compared with the blank group, the model group showed significantly increased inflammatory factor levels (*P* < 0.01). After the intervention of the corresponding Chinese medicine, the inflammatory factor levels were significantly reduced (*P* < 0.01 or *P* < 0.05), and the degree of reduction and drug dose were related, as shown in Figure 4. Next, we also carried out inflammatory factor analysis using immunohistochemistry, and the results were similar to those of ELISA, as shown in Figure 5.

### 3.5. Effects on the SIRT1/TLR4/NF-*κ*B Signaling Pathway

**Figure 6:**
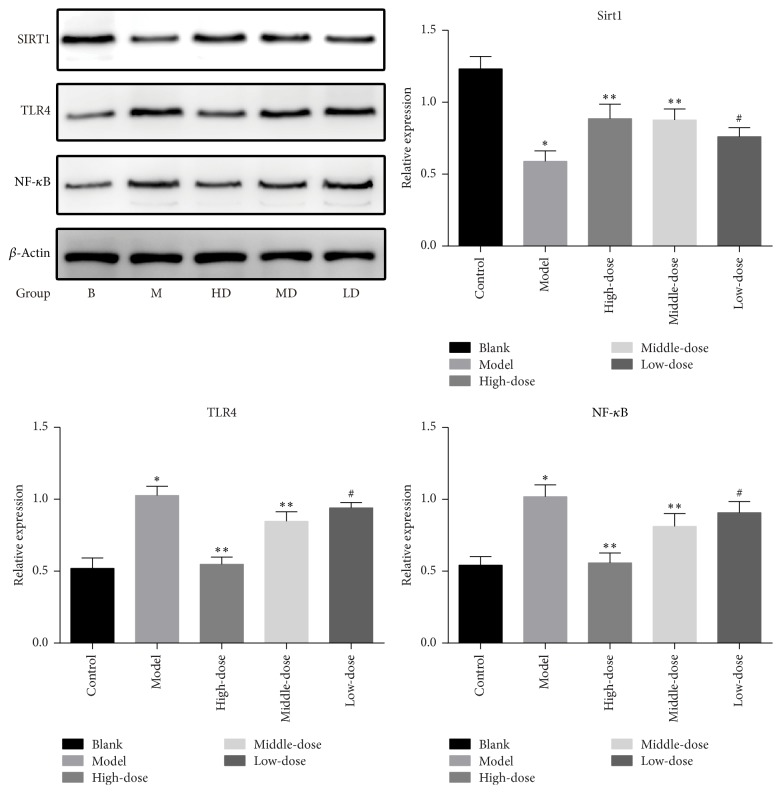
Protein levels after 4 weeks of intervention in the rats (^*∗*^*P* < 0.01 versus the blank group; ^*∗∗*^*P* < 0.01 versus the model group; ^#^*P* < 0.05 versus the model group.

**Figure 7:**
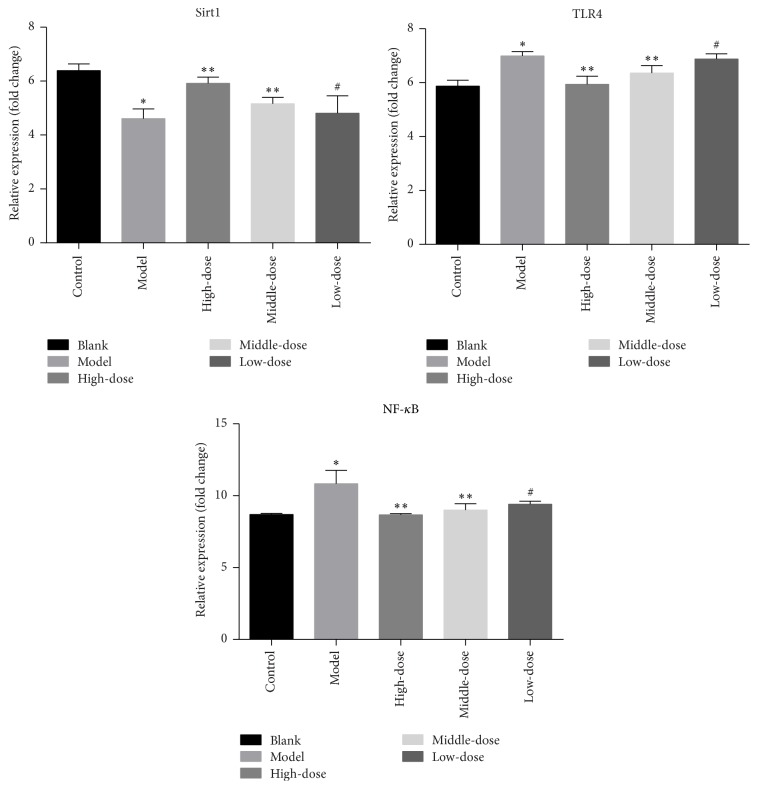
mRNA expression levels after 4 weeks of intervention in the rats (^*∗*^*P* < 0.01 versus the blank group; ^*∗∗*^*P* < 0.01 versus the model group; ^#^*P* < 0.05 versus the model group.

To clarify the inflammatory signaling pathway of drugs, we used western blotting and RT-PCR. The experimental results showed that the model group's SIRT1 expression compared with the blank group was significantly reduced (*P* < 0.01), and TLR4 and NF-*κ*B expression was significantly upregulated (*P* < 0.01). However, the intervention results showed that the level of SIRT1 was increased, and the TLR4 and NF-*κ*B expression was decreased after the intervention with the traditional Chinese medicine (*P* < 0.01 or *P* < 0.05). At the same time, the verification results of PCR were consistent with the results of western blotting. The specific results are shown in Figures 6 and 7.

## 4. Discussion

In this study, we first performed high-performance liquid phase analysis of this formula. The results showed that the components obtained were definitive uridine, inosine, guanosine, safflomin A, and brazilin. Because the composition was relatively simple, the experiments provided favorable conditions. One study showed that* Caesalpinia* and its component brazilin could inhibit the activation of NF-*κ*B signal transduction pathway [9, 16, 17]. Additionally, safflower yellow A (SY), the main active ingredient of the safflower, showed the effects of controlling inflammation and inhibiting the progression of AS in several studies [10–12]. Furthermore, it was shown previously [14, 15, 18, 19] that leeches, which could produce uridine, inosine, and guanosine, could significantly reduce the lipid levels with experimental blood stasis syndrome and significantly control inflammation.

After all of the rats were modeled and subjected to the interventions, endothelial injury and lipid infiltration were observed in the model group after HE staining. Compared with the model group, the HE staining results showed that endothelial injury in each intervention group was improved, indicating that this formula had a definite role in improving endothelial injury. In the meantime, the degree of improvement in endothelial injury and the drug dose were positively correlated.

The blood lipid levels were simpler and more intuitive to investigate, reflecting the progress of the AS reference value [20]. In our study, using Figure 3, we found that the levels of TC, TG, and LDL-C in the model group were significantly higher than those in the blank group, suggesting that the rats in the model group developed AS progression. After the intervention in the corresponding groups, the blood lipid levels in all of the intervention groups had decreased, and the decrease in blood lipid levels was statistically significant. In the test of HDL-C levels, we found that only the results in the high-dose group, compared with those in the model group, showed an increase in the HDL-C levels, and those in the middle-dose and low-dose groups were not significantly improved. According to the results above, we considered, in addition to traditional Chinese medicine with the complete blood lipid-lowering effect, that the inhibition of appetite in the rats might also be a reason for the decreased blood lipid level, an observation that warrants further study.

At present, the inflammatory response theory, as we recognized [21, 22], influenced the pathogenesis of AS. In this study, we used ELISA and immunohistochemistry to detect the relevant inflammatory factors. The results in Figures 4 and 5 showed that IL-1*β*, IL-6, and IL-12 levels in the model group were significantly higher than those in the blank group (*P* < 0.01, *P* < 0.05), and the decreasing trend of inflammatory factors was dose-dependent among the formula intervention group, suggesting that the formula may control the inflammation reaction in endothelial injury, thus affecting the progression of AS.

In recent years, the sirtuin family, also known as the Sir2 (silent information regulator 2) family, has become a research hotspot and was found to be a group III histone deacetylase enzyme in general terms [23]. SIRT1 deacetylates lysine at position 310 of the P65 subunit, which was an important modification of NF-*κ*B, thereby inhibiting NF-*κ*B activity. TLR4 is a transmembrane receptor in the innate immune system that binds nonspecifically to the pathogen-associated molecules and initiates signal transduction [24, 25]. The expression of TLR4 in human umbilical vein endothelial cells (HUVECs) was significantly lower than that in normal HUVECs, but the expression of TLR4 was significantly increased under the stimulation of proinflammatory cytokines in vitro [26–28]. TLR4/NF-*κ*B could induce the synthesis and release of inflammatory cytokines through both MyD88-dependent and MyD88-independent signaling pathways [29]. It was shown [30] that the SIRT1/TLR4/NF-*κ*B pathway may be involved in inflammation to affect the pathogenesis of endothelial injury. We tried to clarify whether the Chinese medicine can improve the inflammatory response through this signaling pathway. The results of western blotting in Figure 6 showed that the expression of SIRT1 in the model group was significantly lower than that in the blank group, while TLR4 and NF-*κ*B were upregulated significantly. The western blotting results of the formula intervention group showed that the SIRT1 level was increased, while the TLR4 and NF-*κ*B levels were decreased. At the same time, we obtained similar PCR results shown in Figure 7, which were matched to those of western blotting. Therefore, we believed that the formula could play a role in this signaling pathway to inhibit endothelial injury and elevate blood lipid levels, thus alleviating the progression of AS.

## 5. Conclusion

The results showed that the Kangshuanyihao formula can inhibit the inflammatory response through the SIRT1/TLR4/NF-*κ*B signaling pathway, thereby improving endothelial injury and lowering the blood lipid levels, to inhibit AS progression. It was also shown that the signaling pathway may be the pathological mechanism of the AS inflammation response of a potential pathway, which will be further confirmed in in vitro experiments.

## Figures and Tables

**Table 1:** 

Genes	Forward	Reverse
Gapdh	5′-TTCAACGGCACAGTCAAGG-3′	5′-CTCAGCACCAGCATCACC-3′
Sirt1	5′-GGGAACCTCTGCCTCATCTAC-3′	5′-GCATACTCGCCACCTAACCT-3′
Tlr4	5′-ATGAGGACTGGGTGAGAAACG-3′	5′-ATGCCAGAGCGGCTACTCA-3′
NF-*κ*B	5′-CATGCGTTTCCGTTACAAG-3′	5′-TGAGGTGGGTCTTTGGTGA-3′
